# Inequality in healthcare use among older people in Colombia

**DOI:** 10.1186/s12939-020-01241-0

**Published:** 2020-10-26

**Authors:** Jorge Garcia-Ramirez, Zlatko Nikoloski, Elias Mossialos

**Affiliations:** 1 Research group GIPSPE, University of Caldas, Manizales, Colombia; 2 Department of Health Policy, The London School of Economics and Political Science, London, UK

**Keywords:** Health equality, Health services, Universal health coverage, Aged, Colombia

## Abstract

**Background:**

Since the early 1990s, Colombia has made great strides in extending healthcare coverage to its population. In order to measure the impact of these efforts, it is important to assess whether the introduction of universal health coverage has translated into equitable access to healthcare in the country, particularly for the elderly. Thus, in this study we assessed the inequality in utilization of health services among elderly patients in Colombia. In addition, we identified the determinants of healthcare utilization.

**Methods:**

We analyzed the 2015 Colombian health, well-being and aging study (SABE). To classify determinants of healthcare use into predisposing, enabling and need factors, we employed the Anderson framework of healthcare utilization. Use of outpatient, inpatient and preventive health services constituted the dependent variables. We performed multivariate logistic regressions, estimated concentration indexes (CI) and performed decomposition analyses of the CIs to determine the contribution of various determinants to inequality of healthcare utilization.

**Results:**

The study sample included 23,694 adults over 60-years-old. Wealth quintile, urban dwelling, health insurance type and multimorbidity predicted the utilization of all types of healthcare services except for hospitalization. Aside from inpatient care, pro-rich inequality in utilization of healthcare services was present. Wealth quintile and type of health insurance were the largest contributors to pro-rich inequality in use of preventive services.

**Conclusions:**

While there has been progress in health insurance coverage for the elderly in Colombia, there are still equality challenges in the delivery of healthcare, especially for preventive and outpatient care. These inequalities are driven by individual characteristics such as wealth, urban residence, type of health insurance carried, and presence of multimorbidity. To address this issue, the Colombian health system should extend health insurance coverage to uninsured populations, as well as reduce barriers of access to healthcare services among poorest and the rural population receiving subsidized insurance.

## Background

### Colombia’s healthcare system

In the 2000 World Health Report, the World Health Organization (WHO) awarded Colombia with the top ranking worldwide for fairness in healthcare finance [1]. This accolade came amidst ravaging civil war throughout the country. Nineteen years later, both Colombia’s social and economic landscapes have improved dramatically. A question arises as to whether the health system in Colombia has progressed at a similar rate [2, 3].

Prior to 1993, Colombia had a National Social Security system which provided insurance to less than 24% of the population and catered mostly to wealthy individuals and formal public employees [4]. The provision of health services under this system occurred through a network of public hospitals [4]. In an attempt to improve healthcare coverage, in 1993, Colombia implemented a major reform to its health system by creating the ‘General System of Social Security in Health’ through the enactment of Law 100 [5]. This reform introduced a mandatory health insurance model based on managed competition between private insurers [5]. By 2017, 94.41% of the population of 45.5 million Colombians was insured. Of those who were insured, 90.24% were covered by one of the two main insurance schemes: the contributory scheme (CS) for the formal workers, or the subsidized scheme (SS) for those without the ability to pay [4, 6]. A smaller proportion (4.17% in 2017) of the population belongs to a special scheme for public teachers, the armed forces, and workers from the state oil company. Despite the major improvement in healthcare coverage in Colombia, there is still a fair portion of the population (5.59% in 2017) who remain uninsured which is comprised of the unemployed, informal workers earning less than minimum wage, and poor families who score above the income threshold for government social benefits under the subsidized scheme [7].

Colombia has made great strides in healthcare financing since reforming its social security system in 1993. In 2015, Colombia spent 6.19% of its GDP on healthcare, and 76% of total health expenditure (THE) was public. In addition, per capita health spending increased from US$360.67 in 2000 to US$382.10 in 2016 (in constant 2000 prices) [8]. Despite these improvements in national health spending, Colombians continue to pay out-of-pocket for healthcare services (18.3% of THE in 2015) [9].

In order to finance the various insurance schemes offered, the Colombian government collects and pools payroll taxes and general taxes [10]. It subsequently allocates resources to 45 competing private insurers based on a per-capita premium adjusted for age, gender, geographic distribution of enrollees, and type of coverage scheme (CS or SS) [10]. Both CS and SS coverage schemes have a different premium base per individual. In 2018, the premiums were US$246.9 and US$220 for CS and SS respectively [5]. Insurers must guarantee the provision of services covered in the health benefits package (HBP), which—since 2012—is identical for both schemes and is updated by the government annually [11, 12].

The 1993 reform of the health system split purchasing and providing functions. Private insurers selectively contract services from public and private health providers. Fees for services and payment schedules are not defined by government and instead are negotiated between insurers and providers. The government is responsible for general stewardship of the system and for the regulation of quality, solvency, and accounting standards of insurers and providers [13, 14]. This fragmentation of the system has arisen as a concern due to its negative impacts, including: lack of coordination between the multiplicity of payers and providers, the burden of administrative bureaucracy to authorize treatments, and deficient organization across levels of care in the territories.

### Equality and utilization – evidence from the literature

Despite substantial population health improvements in the last few decades, equality remains a challenge for many Latin American health systems. Health insurance coverage and income levels are some of the main determinants of access to healthcare within the region [15–17]. Moreover, the utilization of specific health services such as preventive doctor visits, curative doctor visits, and cervical and breast cancer screening is higher among richer segments of the population. Nevertheless, countries such as Colombia and Mexico have shown the largest improvements in access to cancer screenings in Latin America, with levels of service utilization above the regional average and less inequality across income groups than the regional median [18, 19]. Despite significant progress in access to preventive and curative visits in Colombia, the poorest groups still tend to use outpatient services less, while inpatient care has almost an equitable distribution [20].

In considering the progress of health equality in Colombia, it is also important to look beyond insurance coverage and socio-economic status. Experts have found that several other factors contribute to utilization of healthcare services. For example, in 2014, Garcia-Subirats et al. found that having a chronic disease contributed to a higher utilization of preventive and outpatient care [21]. Further, studies have shown that living in a rural region is negatively associated with healthcare use in Colombia [22]. Finally, the complex health insurance and care structure in the country (e.g. deficiency in the coordination across levels of care, the administrative bureaucracy between the multiple payers and providers, and the lack of integration between health actions at the individual and the community level) further impacts upon the equality of utilization [23].

Bearing in mind that the proportion of older adults in Colombia is expected to double by 2050 (from 11 to 25%) and thus health services promoting healthy aging are more important than ever, capturing the use of healthcare in the country—particularly with an equality lens—is an important undertaking. There are no recent studies analyzing equality of healthcare utilization in Colombia, and the research that does exist does not incorporate heavy users (such as older adults) in their analyses. Against this background, this study has two objectives: first, to identify the determinants of healthcare utilization for Colombian elderly patients, and second, to analyze the equality of healthcare utilization among elderly Colombian patients.

Our research question is timely and relevant, particularly as we try to study the determinants of access to healthcare. While existing evidence in Colombia suggests that within country variations in morbidity and mortality have been associated with socio-economic conditions as income, education, gender, and racial disparities, there is little evidence on how some socio-economic factors impact upon access to healthcare [24–27].

## Methods

### Dataset and sample

The SABE study (Health, Aging, and Well-being) is a nationally representative population-based cross-sectional dataset of 23,694 adults over 60-years, which was collected by the Ministry of Health in Colombia in 2015. The survey used in the SABE study followed the conceptual model of active aging and the social determinants of health (see Additional file 1*)* [28].

### Methodology

In order to analyze inequality in utilization of healthcare among the elderly in Colombia, we followed a two-fold approach. In the first instance, we relied on standard multivariate logistic model regression. We followed this with an analysis of inequality in utilization using the standard Concentration Index (CI).

#### Logit model analysis of utilization of healthcare services

In modelling the utilization of healthcare, our main dependent variables encompass six variables which fall within three levels of care. Preventive care (use of pap smear and mammogram for women; prostate cancer lab screening in the last 2 years for men), outpatient care (doctor visit in the last 4 months; visit to any health professional—other than a doctor—in the last year), and inpatient care (hospitalizations in the last year). Consequently, we estimated six separate logit models using each of the variables above as a dependent variable, one at a time.

The basis for the modelling exercise was the Anderson’s behavioral model of health services use; it also enabled the selection of independent variables for the modelling exercise [29]. Andersen establishes that utilization of health services depends on three factors: predisposing, enabling, and need factors. Predisposing factors include individual characteristics present before the occurrence of a disease and are related to demographic conditions. Enabling factors describe the means utilized by individuals in order to access the services they need, such as income. Finally, need factors refer to the health conditions—either perceived or evaluated—requiring medical care.

Andersen’s model groups determinants of access in three major groups (need, predisposing and enabling factors). The model can capture drivers of inequality from both an individual’s and health system’s perspective. There are, however, some limitations associated with the model. For example, the model does not explain the relationship between healthcare utilization and quality of services (health outcomes and patient satisfaction) [29, 30].

Against this background, if we assume a linear model, utilization of healthcare services can be analyzed by regressing the relevant utilization variable (y_i_) on a vector of k medical need indicator variables (x_k_), predisposing factor variables (u_q_), and a set of p enabling factor variables (z_p_) (for example, socioeconomic variables, health insurance, and supply-side variables).

The equation would be as follows:

1 $$ {y}_i^{\ast }=\alpha +{\sum}_k{\beta}_k{x}_{k,i}+{\sum}_q{\delta}_q{u}_{q,i}+{\sum}_p{\gamma}_{p,i}z+{\varepsilon}_i,\kern0.5em \mathrm{with}\kern0.5em \mathrm{i}=1,\dots \mathrm{N} $$

Where α, β, γ_,_ δ= parameters and ε_i_ = error term.

Assuming that y_i_^*^ in equation (A) is a latent variable, the logit model is written as:

$$ \left\{\begin{array}{c}1\  if\kern0.5em {y}_i^{\ast }>0\\ {}0, otherwise\end{array}\right. $$

Our dependent variables encompass three levels of healthcare utilization: (i) preventive care (e.g. screening activities); (ii) outpatient care (curative and rehabilitation services provided by a healthcare professional at the primary level (both acute and chronic care), that do not require hospital stay) and (iii) inpatient care (curative and rehabilitation services provided at a hospital, and requiring overnight stay, usually for high-complex care).

To assess medical need factors, multimorbidity and self-rated health (SRH) were used as proxies. The SABE study asked participants if they had ever been diagnosed as having high blood pressure, diabetes, osteoarthritis, ischemic heart disease, cerebrovascular disease, chronic respiratory disease or cancer. Therefore, a categorical variable was created encompassing the following: no presence of chronic disease, presence of one chronic disease, and presence of two or more chronic diseases. SRH measured the subjective health experience of individuals by answering the question: “In general, how would you rate your health in the last 30 days?” Based on this question, a dummy variable was created by taking a value of 1 for answers very good, good, and fair and a value of 0 for answers poor and very poor. We also included as need factors, four variables assessing the functional impairment of individuals: the Barthel index [31], any walking impairment, need of walking help, and presence of any amputation. The Barthel index is a geriatric score evaluating the level of dependency giving a score to each individual. We classified individuals as independent, with mild dependency, with moderate to severe dependency and with total dependency. For any walking impairment, individuals were asked if they found difficult walking 400 m. Individuals were subsequently classified as having no difficulty, mild difficulty, somewhat or significant difficulty. For assessing the need of ‘any walking help’, individuals were also asked if they needed any help walking 400 m. The variable took the value of 1 if an individual needed any walking help and zero if they didn’t need any. The variable ‘any amputation’ took the value of 1 if the individual had any limb amputation and zero if they didn’t have any.

Predisposing factors included age, gender, marital status, level of education, belonging to an ethnic minority and displacement. Four, five-year groups represented the age variable: 60–65, 66–70, 71–74 and 75 and older. A dummy variable capturing ethnicity was created, which took the value of 1 if the respondent belonged to any ethnic minority (mixed, black, islander, palenquero or indigenous), and 0 otherwise. Marital status was proxied by a dummy variable that took a value of 1 (being married/cohabiting and divorced/widowed) or 0 (otherwise). Level of education was captured by a categorical variable among four options: no formal education, primary school, secondary school, or technical education and above. In addition, a dummy variable for displacement was created which took a value of 1 if the respondent was displaced and 0 otherwise.

Enabling factors included wealth index, area of residence, type of health insurance, geographic region and receiving a pension. We created a wealth index for all participants as a proxy of their socio-economic level (see Additional file 2*)* and classified participants into five different wealth quintiles. Based on the area of residence, we created a dummy variable which took a value of 1 if the respondent lived in an urban area and 0 if they lived in a rural setting. The health insurance type was captured by a categorical variable consisting of four categories: subsidized, contributory, special schemes, and uninsured; and the geographic region variable corresponded to the six regions of the country in which the survey aggregated the participants. Given that the survey did not include supply-side variables (e.g. density of doctors or nurses), this variable was used as a proxy for regional variation in supply-side healthcare variables. Finally, a dummy variable which captured whether the respondent received a pension was included.

We used standard weights in the analysis and reported the results as odds ratios. In addition, we reported the standard Wald (Chi2 test) and the log likelihood. All analyses were conducted in STATA version 14.0.

#### Concentration index for inequality of utilization

We coupled the logit model exercise with a calculation of concentration index (CI) and decomposition of CI in order to quantify the degree of equality in the utilization of health services and the extent to which each of the three groups of variables above (medical need, predisposing, and enabling) contributed to the inequality of utilization [32].

CI is defined with reference to the concentration curve. It is twice the area between the concentration curve and the line of equality (the 45-degree line). Concentration curves plot the specific health variable in the y–axis against the percentage distribution based on a wealth measure in the x–axis. Therefore, CI takes a value ranging from (− 1, 1) where negative values express pro-poor concentration and positive values express pro-rich concentration. Equation 2 presents the general model for CI:

2 $$ C=\frac{2\ }{\mu }\ {\mathit{\operatorname{cov}}}_w\left({y}_i,{r}_i\right) $$

Where *C* is the CI, *y*_i_ is the measure of utilization of healthcare services, μ is its mean, and *r*_*i*_ is the rank distribution of an individual *i* according to his wealth index.

The decomposition of the CI shows the contribution of the independent variables in the logit model to the distribution (inequality) of health services based on the wealth rank of the population. It provides more detailed information and raises potential areas for policy intervention. We relied on methodology for the decomposition analysis that used a probit model and its ‘partial effects’ (i.e. the effects of an individual independent variable, ceteris paribus) as eq. 3 depicts:

3 $$ E\left({y}_i|{x}_i\right)=G\ \left({\sum}_k{\beta}_k{x}_k^i\right) $$

where G represents the functional form for a nonlinear model. As proposed by van Doorslaer et al. [32], we have restored the mechanics of the decomposition framework by replacing the βk parameters in the equation with the βmk parameters, where the βmk represent the partial effects of the x (the determinants of y) in the linear approximation of the non-linear model expressed by Eq. 4:

4 $$ {y}_i={\sum}_k{\beta}_k^m{x}_k^i+{\mu}_i $$

Accordingly, we conducted a decomposition of the socio-economic related inequality affecting healthcare utilization.

## Results

### Descriptive statistics for models of utilization of healthcare

Table 1 shows the summary statistics for the full sample and the subgroups of both, the insured and the uninsured population. Among the participants, 60.3% were aged 60 to 70 years old, 97.8% had any type of health insurance, and 75.7% suffered from one chronic disease or more. The proportion of uninsured participants in SABE (2.2%) was lower than that in the general population (3.2% in 2015) [6]. The uninsured belong to younger age groups, mostly do not cohabitate, tend not to have pensions, are urban dwellers (70.4%), and are concentrated in the poorest wealth quintiles (40% in the poorest quintile compared to 7.29% in the richest one). Furthermore, 57.9% of the uninsured had at least one disease. A large majority of the insured have no or little functional impairment. The table also includes the standard test for statistical significance between the two groups (Table 1).

**Table 1:** Summary statistics for independent variables (weighted)

	Total sample ***N*** = 23,694		Insured ***n*** = 23,152		Uninsured ***n*** = 518		***P*** value¬
Variable	n	%	n	%	n	%	
**Predisposing factors**							
Age							
60–65	9010	38.03	8695	37.55	306	59.12	< 0.001
66–70	5268	22.23	5176	22.36	87	16.81	
71–75	3943	16.64	3891	16.81	48	9.29	
76–80	2814	11.88	2772	11.97	39	7.54	
80+	2659	11.22	2619	11.31	37	7.24	
Male	10,776	45.48	10,457	45.17	308	59.49	< 0.001
Marital status^a^	12,392	52.32	12,180	52.61	205	39.63	< 0.001
Education^a^							
No formal education	3919	16.61	3776	16.37	139	26.99	< 0.001
Primary school	12,599	53.39	12,353	53.57	232	45.15	
Secondary school	4521	19.15	4395	19.06	121	23.44	
Technical or higher	2562	10.85	2536	11.00	23	4.42	
Ethnicity^a^	3736	20.95	3625	20.76	107	29.52	0.037
Displacement^a^	3631	15.33	3521	15.21	107	20.61	0.270
**Socioeconomic factors**							
Wealth quintile							
Poorest	4739	20.00	4527	19.55	207	40.03	< 0.001
2nd poorest	4739	20.00	4616	19.94	118	22.85	
Middle	4738	20.00	4653	20.10	81	15.63	
2nd richest	4739	20.00	4661	20.13	74	14.20	
Richest	4738	20.00	4696	20.28	38	7.29	
Geographic region in Colombia							
Bogota	4032	17.02	3970	17.15	58	11.15	0.028
East	4248	17.93	4109	17.75	135	26.02	
Orinoquia and Amazon	328	1.38	310	1.34	17	3.37	
Atlantic	4527	19.11	4392	18.97	130	25.19	
Central	6400	27.01	6313	27.27	80	15.51	
Pacific	4160	17.56	4059	17.53	97	18.75	
Urban	18,524	78.18	18,141	78.35	365	70.40	< 0.001
Type of health insurance^a^							NA
Uninsured	518	2.19	NA		NA		
Subsidized	11,104	46.91	NA		NA		
Contributory	11,574	48.90	NA		NA		
Special	474	2.00	NA		NA		
Receives a pension^a^	6754	28.52	6741	29.12	10	1.90	< 0.001
**Need factors**							
Multimorbidity^a^							
No diseases	5659	24.27	5440	23.88	211	42.07	< 0.001
1 disease	7017	30.10	6885	30.22	124	24.70	
Multimorbid (2 diseases or more)	10,637	45.63	10,457	45.90	167	33.23	
Self-rated health^a^	9741	51.27	9527	51.24	209	52.56	0.020
Barthel Index (dependency)							
Independent	18,666	78.78	18,223	78.71	423	81.74	0.005
Mild dependency	2249	9.49	2213	9.56	34	6.60	
Moderate to severe dependency	2616	11.04	2554	11.03	59	11.40	
Total dependency	163	0.69	162	0.70	1	0.25	
Walking impairment^a^							
No impairment	15,835	66.90	15,422	66.68	398	76.89	< 0.001
Mild difficulty	3503	14.80	3435	14.85	65	12.59	
Somewhat/Very difficult	4332	18.30	4274	18.48	54	10.52	
Walking help^a^	19,896	84.48	19,411	84.35	465	90.38	0.001
Amputation	486	2.05	476	2.06	9	1.75	0.719

¬ *P* value for Pearson chi square test comparing insured individuals and uninsured individuals

^a^Number of observations were not equal to the 100% of the total sample nor for subsamples by insurance categories. Observations N (%) were equal to: marital status: 23684 (99.9), education (99.5), ethnicity:17893 (77.7), displacement:23689 (99.9), Type of health insurance:23670 (99.9), receives a pension: 23680 (99.9), multimorbidity: 23312 (98.6), self-rated health: 18999(80.2), walking impairment: 23672(99.9), walking help: 23560 (99.4)

*NA* Not applicable

The healthcare service with the highest usage was a physician visit in the last 4 months (74.9%), while only 12.9% of the respondents reported being hospitalized in the last year. The uptake of preventive screening services for men and women ranged from 41 to 53.6%—a fairly low participation percentage when considering the public health importance of those diseases screened (breast, cervical, and prostate cancer). Finally, a higher share of insured participants (compared to uninsured) have accessed the services featured in Table 2.

**Table 2:** Summary statistics for dependent variables (weighted)

	Total sample ***N*** = 23,694		Insured ***n*** = 23,152		Uninsured population ***n*** = 518		***P*** value¬
Variable	n	%	n	%	n	%	
Any visit to a health professional in the last year	10,895	45.98	10,763	46.49	121	23.38	< 0.001
Any doctor visit in the last 4 months^a^	17,738	74.93	17,559	75.92	161	31.00	< 0.001
Any hospitalization in the last year^a^	3061	12.92	3009	13.00	49	9.40	< 0.001
Use of pap smear in the last 2 years^a^	7256	53.63	7165	53.86	84	40.07	< 0.001
Use of mammogram in the last 2 years^a^	5547	40.96	5498	41.29	45	21.40	< 0.001
Prostate cancer screening in the last 2 years^a^	4624	46.11	4563	46.99	51	16.57	< 0.001

¬ *P* value for Pearson chi square test comparing insured individuals and uninsured individuals

^a^Number of observations were not equal to the 100% of the total sample nor for subsamples by insurance categories. Observations for total population N (%) were equal to: any doctor visit: 23672 (99.9), any hospitalization: 23689(99.9), use of pap smear: 13530 (54.3), use of mammogram:13541 (54.3), prostate cancer screening: 10027(45.3)

### Logit model analyses

Table 3 shows the results of the regression analyses. Our findings suggest that the enabling factors bear the highest weight in explaining the variation in utilization of healthcare among elderly Colombians. Specifically, wealth quintile, residing in an urban area and type of health insurance were the variables most significantly associated with the utilization of every healthcare service aside from hospitalization. Older adults from the wealthiest quintile were between 1.5 and 3.5 times more likely to use an outpatient or preventive service compared to the poorest quintiles. Moreover, older adults in urban areas had between 1.5 and 1.9 times the odds of using outpatient care and inpatient care compared to their rural counterparts. Having health insurance is associated with higher utilization of both preventive and outpatient care. When compared to the uninsured, participants with subsidized healthcare were 6.3 times more likely to have visited a doctor in the past 4 months, and 2.3 times more likely to have been screened for prostate cancer; participants with contributive healthcare insurance were 6.4 and 5.5 times more likely respectively. When comparing healthcare utilization among insured individuals, respondents with contributive health insurance were more likely to use preventive care than subsidized ones (1.7 times more likely for the use of Pap smear; 2.2 times more likely for the use of mammogram; and 2.3 times more likely for prostate cancer screening).

**Table 3:** Binary logistic regressions results for healthcare utilization

		Any visit to a health professional in the last year		Any doctor visit in the last 4 months		Any hospitalizations in the last year		Use of pap smear in the last 2 years		Use of mammogram in the last 2 years		Prostate cancer screening in the last 2 years	
**Variable**	**Categories**	OR	SE	OR	SE	OR	SE	OR	SE	OR	SE	OR	SE
**PREDISPOSING FACTORS**													
*Age*	60 to 65^c^												
	66 to 70	0.940	0.104	0.915	0.131	0.960	0.140	0.692^b^	0.092	0.710^a^	0.105	1.332	0.207
	71 to 75	0.932	0.107	1.182	0.151	1.108	0.170	0.257^b^	0.036	0.404^b^	0.063	1.116	0.184
	76 to 80	0.859	0.112	1.283	0.194	0.982	0.178	0.235^b^	0.041	0.469^b^	0.101	1.514^a^	0.282
	> 80	0.666^b^	0.095	1.134	0.192	1.071	0.230	0.130^b^	0.028	0.238^b^	0.060	0.771	0.160
*Gender*	Male^c^												
	Female	0.876	0.077	0.817	0.087	1.268	0.165	NA		NA		NA	
*Marital status*	Not married^c^												
	Married or cohabiting	0.925	0.082	1.011	0.120	1.082	0.130	1.366^b^	0.142	1.336^a^	0.153	1.403^b^	0.182
*Education*	No formal education^c^												
	Primary	1.193	0.143	1.152	0.189	1.119	0.187	1.124	0.155	1.269	0.219	1.203	0.214
	Secondary	1.904^b^	0.275	1.280	0.252	1.271	0.251	0.981	0.168	1.469	0.295	1.498	0.316
	Technical or above	3.178^b^	0.649	1.247	0.369	1.255	0.325	1.006	0.265	2.805^b^	0.854	0.835	0.213
*Ethnic minority*	Not belonging to an ethnic minority^c^												
	Belonging to an ethnic minority	1.113	0.099	0.954	0.096	1.092	0.127	0.839	0.118	0.882	0.120	1.343^a^	0.176
*Displacement*	Not being displaced^c^												
	Being displaced	0.862	0.099	1.087	0.126	0.952	0.150	1.124	0.176	0.936	0.153	0.949	0.144
**ENABLING FACTORS**													
*Wealth quintile*	Poorest ^c^												
	2nd poorest	1.466^b^	0.155	1.304^a^	0.157	0.955	0.133	1.589^b^	0.235	1.123	0.172	1.286	0.219
	Middle	1.436^b^	0.162	1.238	0.160	0.999	0.161	1.505^b^	0.239	1.545^b^	0.253	1.308	0.237
	2nd richest	1.740^b^	0.225	1.328	0.207	1.399^a^	0.215	1.695^b^	0.289	1.630^b^	0.291	1.707^b^	0.334
	Richest	2.370^b^	0.376	1.696^a^	0.348	0.910	0.180	3.357^b^	0.666	2.095^b^	0.473	1.573^a^	0.342
*Geographic region*	Bogota^c^												
	Oriental	0.994	0.157	1.000	0.211	1.202	0.253	0.827	0.165	1.273	0.288	1.550	0.349
	Orinoquia and Amazonia	1.207	0.306	1.592	0.448	1.549	0.698	1.509	0.438	0.941	0.283	1.678	0.642
	Atlantico	0.878	0.126	1.472^a^	0.258	1.364	0.243	1.151	0.196	1.236	0.227	1.410	0.304
	Central	0.659^b^	0.093	0.986	0.185	1.351	0.242	1.052	0.172	1.298	0.252	1.158	0.241
	Pacifica	1.012	0.144	1.181	0.224	1.128	0.216	1.095	0.199	1.018	0.209	1.193	0.268
*Area of residence*	Rural^c^												
	Urban	1.658^b^	0.194	1.501^b^	0.196	1.468^a^	0.223	1.499^b^	0.227	1.880^b^	0.332	1.767^b^	0.287
*Type of health insurance*	Uninsured^c^												
	Subsidized	1.808^a^	0.508	4.967^b^	1.263	1.186	0.414	1.744	0.593	1.890	0.638	2.345^b^	0.862
	Contributory	2.011^a^	0.575	6.376^b^	1.673	1.340	0.479	2.839^b^	0.987	4.210^b^	1.447	5.47^b^	2.055
	Special^d^												
*Type of health insurance (sub-analysis)*	Subsidized^c^												
	Contributory	1.120	0.104	1.286^a^	0.147	1.122	0.145	1.654^b^	0.204	2.248^b^	0.275	2.343^b^	0.331
*Receives a pension*	No^c^												
	Yes	1.256^a^	0.143	1.113	0.169	0.638^b^	0.100	1.157	0.181	0.869	0.146	1.316	0.213
**NEED FACTORS**													
*Multimorbidity*	No diseases^c^												
	One chronic disease	1.459^b^	0.157	2.382^b^	0.264	1.450^a^	0.258	1.658^b^	0.237	1.363^a^	0.203	1.771^b^	0.260
	Multimorbid	1.945^b^	0.207	3.987^b^	0.528	2.599^b^	0.432	1.569^b^	0.216	1.498^b^	0.225	2.307^b^	0.331
*Self-rated health*	Bad health^c^												
	Good health	0.895	0.074	0.574^b^	0.053	0.616^b^	0.078	1.125	0.117	1.180	0.134	0.966	0.115
*Barthel index (dependency)*	Independent^c^												
	Mild dependency	1.335^a^	0.190	1.202	0.242	1.760^b^	0.304	0.955	0.165	0.962	0.167	1.072	0.248
	Moderate to severe dependency	1.188	0.200	1.204	0.250	1.490	0.325	0.814	0.160	0.671	0.159	0.980	0.258
	Total dependency	2.246	1.857	1.355	1.373	1.200	1.167	0.173	0.189	0.011^b^	0.015	No observations	
*Walking Impairment*	No impairment^c^												
	Mild difficulty	0.987	0.120	1.106	0.149	1.044	0.169	0.994	0.132	1.123	0.163	0.992	0.197
	Somewhat/Very difficult	1.060	0.119	1.420^a^	0.207	1.047	0.163	0.832	0.118	0.913	0.138	1.154	0.226
*Walking help*	No help needed^c^												
	Help needed	1.268	0.185	1.150	0.234	0.653	0.152	1.242	0.213	1.041	0.206	1.103	0.279
*Amputations*	No amputations^c^												
	Any amputation	1.881	0.646	0.532	0.213	2.027^a^	0.637	0.803	0.414	0.433	0.252	0.658	0.248
***Statistical parameters***	Observations	17,459		17,444		17,455		9735		9741		7663	
	Wald (Chi2) test	400.2		392.26		202.48		432.3		361.3		280	
	Log Likelihood	−2,495,693.8		−1,963,778.3		−1,280,073		−1,219,592.2		−1,251,254.3		−1,127,363.8	
	*p*-value	< 0.001		< 0.001		< 0.001		< 0.001		< 0.001		< 0.001	

^a^Statistical significance at 5% level ^b^Statistical significance at 1% level ^c^Baseline variable for comparison ^d^excluded from the analysis

*OR* Odds Ratio, *SE* Standard error, *NA* Not applicable

Finally, we found some evidence for association between need and utilization of healthcare services. For example, multimorbid participants have 3.9 times higher odds of visiting a doctor in the last 4 months and 2.6 times higher odds of having been hospitalized in the last year than those without any disease. The rest of the need variables do not show statistically significant link with our main dependent variables

### Concentration index and decomposition analysis for inequality of healthcare utilization

Table 4 presents the results of the CI analysis. We found a consistent pro-rich inequality in utilization of most health services. The most unequal service was the use of mammogram, followed by visiting a health professional in the last year. Finally, we did not find evidence for inequality in utilization of hospital care.

**Table 4:** Concentration indices for healthcare utilization

Variable	CI	SE	t (C)	***P***-value	95% Confidence Interval	
Any visit to a health professional in the last year	0.138	0.011	12.040	< 0.001	0.115	0.160
Any doctor visit in the last 4 months	0.038	0.006	6.230	< 0.001	0.026	0.051
Any hospitalization in the last year	−0.010	0.024	−0.420	0.672	−0.058	0.037
Use of pap smear in the last 2 years	0.112	0.013	8.950	< 0.001	0.087	0.137
Use of mammogram in the last 2 years	0.159	0.019	8.350	< 0.001	0.122	0.196
Prostate cancer screening in the last 2 years	0.131	0.017	7.510	< 0.001	0.097	0.165

*CI* Concentration index, *SE* Standard error

Table 5 depicts a summary of the contributions to the CI for each of the predisposing, enabling, and need factors. We present the results for three of the six models (visit to a health professional, use of mammogram, and prostate cancer screening) as they showed a constant association between predisposing, enabling, and need factors with utilization of health services. These models cover both outpatient and preventive care (Additional file 3 presents the results of the decomposition analysis for the remaining three models).

**Table 5:** Decomposition of concentration indices for healthcare utilization

Variable	Any visit to a health professional in the last year				Use of mammogram in the last 2 years				Prostate cancer screening in the last 2 years			
	Elasticity	CI	Contribution to CI (%)	*p*-value	Elasticity	CI	Contribution to CI (%)	*p*-value	Elasticity	CI	Contribution to CI (%)	*p*-value
**PREDISPOSING FACTORS**												
Age (above 65 years)^a^	− 0.109	−0.015	1.223%	0.046*	−0.438	−0.015	4.377%	< 0.001**	−0.084	− 0.015	0.954%	0.711
Being male	−0.025	−0.024	0.427%	0.230	NA				NA			
Married/cohabiting	−0.022	0.055	−0.866%	0.378	0.077	0.055	2.734%	0.012**	0.080	0.055	3.235%	0.005*
Education (Primary education or above)^a^	0.222	0.138	22.321%	< 0.001**	0.212	0.138	19.030%	0.001**	−0.035	0.138	−3.601%	0.485
Belonging to an ethnic minority	0.010	−0.138	−0.996%	0.056	−0.009	− 0.138	0.779%	0.366	0.027	−0.138	−2.785%	0.025*
Being displaced	−0.009	− 0.207	1.411%	0.239	−0.008	− 0.207	1.123%	0.645	−0.003	− 0.207	0.490%	0.404
**ENABLING FACTORS**												
Wealth quintile (2nd poorest and above)^a^	0.312	0.267	60.495%	< 0.001**	0.276	0.267	47.905%	< 0.001**	0.140	0.267	27.591%	0.067
Geographic region (living outside Bogota)^a^	0.006	−0.045	−0.207%	0.045*	0.002	−0.045	− 0.044%	0.842	0.068	−0.045	−2.241%	0.933
Area of residence (urban)	0.180	0.020	2.595%	< 0.001**	0.242	0.020	3.108%	< 0.001**	0.197	0.020	2.876%	< 0.001**
Type of health insurance (insured)^a^	0.235	0.053	9.102%	0.101	0.832	0.053	28.803%	< 0.001**	0.679	0.053	26.672%	< 0.001**
Receives a pension	0.031	0.307	6.904%	0.047*	−0.025	0.307	−4.956%	0.464	0.036	0.307	8.235%	0.086
**NEED FACTORS**												
Multimorbidity (one chronic disease or more) ^a^	0.198	0.044	6.402%	< 0.001**	0.105	0.044	3.033%	0.043*	0.234	0.044	7.668%	< 0.001**
Self-rated health (good health)	−0.031	0.048	−1.108%	0.097	0.046	0.048	1.439%	0.131	−0.006	0.048	−0.206%	0.711
Barthel Index (not being independent) ^a^	0.175	−0.011	−1.432%	0.068	−0.505	−0.011	3.692%	0.051	−0.360	−0.011	2.990%	0.897
Walking impairment (having any impairment) ^a^	0.019	−0.021	−0.283%	0.950	−0.040	− 0.021	0.541%	0.882	0.046	−0.021	−0.698%	0.604
Walking help	0.097	0.008	0.570%	0.053	0.019	0.008	0.098%	0.738	0.041	0.008	0.246%	0.644
Amputations	0.006	0.144	0.635%	0.061	−0.009	0.144	−0.834%	0.190	−0.004	0.144	−0.392%	0.321
Number of observations	17,535				9791				7707			
Total contribution (statistically significant factors)	99.73%				108.99%				38%			

* Statistical significance at 5% level ** Statistical significance at 1% level. *CI* Concentration index, *NA* Not applicable

^a^categorical variable that was recoded as a dichotomous variable for the decomposition analysis. The text in parenthesis indicates the new category used

Wealth quintile and type of health insurance contributed most to the pro-rich inequality of utilization of preventive services (ranging from 27.6 to 47.9% for wealth quintile and 26.7 to 28.8% for the type of health insurance), while education also contributed (19%) to the pro-rich inequality in utilization of mammograms. Urban residence contributed to the inequality in every model of utilization (except for inpatient care), although with a smaller magnitude: the contribution of the urban residence variable was less than 4% in the models (2.6 to 3.8% for outpatient care, and 2.2 to 3.1% for preventive care).

## Discussion

In this paper, we assessed the equality of utilization of a set of healthcare services among Colombian elderly patients. Firstly, we found that enabling factors (mainly socio-economic standing) explained most of the variation in utilization of health services. The wealthiest individuals were more likely to use preventive and outpatient care compared to those belonging to lower socioeconomic groups.

These findings are consistent regardless of the econometric technique used (logit models or CI). In fact, every model of utilization of health services showed a pro-rich inequality, except for inpatient care. More specifically, visiting a doctor had a mild pro-rich inequality (below 0.1), while preventive services and visiting any health professional had a moderate pro-rich inequality (between 0 and 0.3). These results could reflect the fact that better-off individuals might have increased awareness and demand for accessing preventive care and services beyond medical care, they could also have the resources to access them. These results are in line with the findings of other studies in Latin America quantifying income-related inequalities in healthcare utilization for the general population [16, 17, 33, 34]. For instance, CI for doctor visits in Brazil was 0.398 in 2013, while in Mexico it was 0.021 in 2013 [16, 33]. In addition, Ruiz-Gomez et al. found a CI of 0.091 for preventive doctor visits in 2008 in Colombia, which was higher than the CI of 0.038 we found for that of the elderly population in this study [20]. Furthermore, there is ample evidence from the Latin American region showing the link between socio-economic status and healthcare utilization [35–37].

Wealth can facilitate access to healthcare for individuals through a variety of mechanisms. Wealth provides the economic resources for patients to seek alternative private providers when their health insurance funds deny services. Moreover, wealth is generally linked to higher education, which implies that the wealthiest individuals are enabled to navigate the Colombian health system in the case they face access barriers. Finally, wealthy individuals have greater health awareness (also, in part, due to higher education levels), which increases their demand for healthcare, especially for preventive care.

We did not find inequality of access to inpatient care based on wealth (logit model or concentration index). This result is aligned with the findings of Ruiz-Gomez et al. in Colombia and Dmytraczenko and Almeida, for 7 Latin American countries, where most hospital services were equally distributed among the rich and the poor [19, 20]. This finding could reflect an adequate protection of older adults in Colombia for high-complex acute care or the lack of proxy variables for acute care need in the survey. Finally, there may also be a reverse causality in that better off people may have better self-rated health as they may have better and more frequent access to preventative and curative health services.

We found socio-economic status to be one of the main determinants of the type of healthcare service that patients used. Specifically, our analysis found the highest magnitude of pro-rich inequality in the utilization of preventive care (CI of 0.159 for mammogram, and 0.131 for prostate cancer screening). These findings are consistent with the results of Cookson et al., who found that in England, the poorest individuals have higher utilization rates of publicly-funded healthcare services compared to richer people, while the wealthiest groups have higher utilization rates of preventive services and access to healthcare at earlier stages of illness [38]. Pro-rich inequities in use of preventive care can often lead to substantial pro-rich inequalities in population health outcomes.

The type of health insurance an individual is covered by affects his/her healthcare usage. Our study results found higher odds of healthcare utilization by insured elders compared to uninsured ones, and likewise, by contributory elders compared to subsidized ones. The mechanisms by which the health insurance scheme produce inequality may be related to the design of the Colombian health system. For example, the uninsured population only has access to emergency care. Additionally, the selection mechanism (means-test threshold) for subsidized health insurance in Colombia may leave the near-poor, who fall above the threshold but do not have the financial means to pay for insurance, without health coverage.

Furthermore, while the pooling of financial resources is centralized by the government, and—since 2012—the benefits package is identical for both the CS and SS schemes, the per capita premium differs. This leads to a structural underfinancing of the subsidized scheme compared to the contributory one, affecting the stability of the insurance funds and therefore their capacity to contract services. Health insurance funds have autonomy for purchasing services from providers by setting rates and payment mechanisms. More specifically, insurance funds under the contributory scheme may set rates and payment mechanisms that are more attractive to health providers than the funds under the subsidized scheme. Therefore, contributory funds may offer better contracts to providers compared to subsidized funds, which in turn could lead to prioritization of care for CS patients. Indeed, this is further corroborated by our findings on outpatient and preventive care.

Evidence from our study suggests that supply-side characteristics, such as urban dwelling, contribute to higher utilization of healthcare. In Colombia, health providers, including physicians dedicated to specialty and/or complex care and health professionals other than doctors (i.e. physiotherapists, nutritionists, optometrists), are concentrated in urban areas. Rural areas are often only served by public providers. Therefore, rural residents may lack choice in accessing service providers, and the public providers they do have access to may not offer the full spectrum of health services they need.

Finally, our results correspond to the design of the health system in Colombia. Under a managed competition model, there are different contracting arrangements and models of care between insurance funds and providers resulting in the implementation of mechanisms to limit the use of services, such as pre-authorization requirements, frequent denial of services, and fragmentation in the continuum of care, de facto creating barriers to accessing healthcare [39, 40]. In addition, there are supply and demand side barriers. Supply-side barriers include the geographic inequalities in service delivery, shortage of specialized services and organizational barriers such as bureaucracy, waiting times, and low quality of services [41], while demand-side barriers include financial barriers to access services, health literacy, and lack of knowledge of patient’s rights and processes to access care [42].

Consistent associations between predisposing factors and healthcare utilization were not found in this study. For the case of education, the lack of association reported in our study is contrary to findings from international literature. A possible explanation could reside in the sample characteristics, as only 10.9% of the respondents had a degree above secondary school. This is not just a pattern of the sample itself, but one of a transitioning economy, such as Colombia, where younger generations are achieving higher educational levels than older ones. Although it was expected that displacement would predict healthcare utilization due to the long history of armed conflict in Colombia [43], this variable was not correlated with any of the models in our analysis. This finding suggests that displaced people in Colombia have healthcare protection and confirms the positive impact of targeted public policy programs: once the government identifies an individual as displaced, they receive immediate health coverage by the subsidized scheme. Colombia has implemented differential public health strategies for the most vulnerable, and has prioritized access to healthcare without any restriction to older and displaced individuals, as stated in the national aging policy and the national public health plan [44–46].

Using a recent, large, and nationally representative dataset, our study provides updates to previous equality studies in Colombia. To the best of our knowledge, this is the first study quantifying equality in healthcare utilization among older adults in Colombia. The primary strength of this study was that it combined two methodological approaches for equality measurement (logit models and concentration indices), which were found to be complementary. Additionally, this study used a wealth index as a proxy of socioeconomic level, which provides a more robust measure of socioeconomic position than traditional measures such as income or socio-economic strata. Finally, we analyzed a comprehensive set of services in the care continuum (preventive care and curative care; outpatient and inpatient care), which also adds to the strength of this study.

### Limitations

This study had several limitations. First, data from a cross-sectional population survey restricts the inferences at the causal level. Second, self-reported data for access to healthcare could lead to recall bias with a subsequent underestimation of utilization, which in turn could affect the magnitude of the associations analyzed in the CI and decomposition models (as briefly mentioned in the discussion, better off people may report better self-rated health because they may have better and more frequent access to healthcare). Third, the data was disaggregated at the regional level, which prevented us from assessing supply-side factors at the district level. As these factors represent fixed effects, they are highly correlated with the geographic region variable we already employed in the analyses. Fourth, the analyses did not include unmet needs variables, i.e. individuals who needed health care in a specific point of time and did not receive it because of financial, geographical, and supply-side barriers. Therefore, the levels of inequality found may be underestimated. Finally, while we try to account for as many need variables as possible, the survey does not include questions on acute care need which might have a bearing on the results on the inequality in access to inpatient care.

## Conclusions

The findings of our study suggest that despite Colombia’s progress in extending health insurance coverage to its population, an equal provision of healthcare services to older adults is not guaranteed under the current system. Therefore, although the country’s economic and social landscape has changed dramatically over the last decade, there are still equality challenges in the delivery of healthcare for the elderly population—especially for preventive and outpatient care—which are driven by individual characteristics such as wealth, urban residence, type of health insurance carried, and presence of multimorbidity. To address these issues, the Colombian health system should start by extending health insurance coverage to uninsured populations, who are the most vulnerable. Subsequently, service delivery programs and efforts to reduce access barriers should be targeted towards the poorest and those groups receiving subsidized insurance in rural populations. Finally, preventive care efforts should be strengthened and promoted—especially for women and for the poorest population groups—to improve health outcomes and overall population health.

Future studies would benefit from contrasting these results with other domains of Colombia’s health system performance (i.e. quality of health services and the distribution of health outcomes among older populations). Additionally, assessing equality over time through longitudinal studies might help to better understand the impact of policies in the healthcare sector. Although not directly generalizable, the results of this study may shed light on potential health equality intervention target areas for other Latin American countries with similar health system structures.

## Supplementary information

**Additional file 1.** Description of the SABE study.

**Additional file 2.** Construction of wealth index (asset index) in the study.

**Additional file 3.** Additional decomposition analyses of concentration indices.

## Data Availability

The data and all background materials are available upon request.
