# Supplementary material for: Inequality in healthcare use among older people in Colombia
Source: Int J Equity Health. 2020 Oct 26;19:168. doi: 10.1186/s12939-020-01241-0 (PMC7646194; doi:10.1186/s12939-020-01241-0)
Supplement: Supplementary file 1 — Additional file 1. Description of the SABE study. [file 12939_2020_1241_MOESM1_ESM.docx]

**Additional file 1- Description of the SABE study**

The SABE study (Salud, Bienestar y Envejecimiento - *Health, Well-being and ageing*) was the first national health survey in Colombia assessing the health of older adults. The University of Valle and the University of Caldas executed the survey in partnership with the Colombian Ministry of Health as part of its national health surveys system. Data was collected between 2014 and 2015, and the results and dataset were published in May 2016 (1).

The study included adults aged 60-years or older and excluded institutionalized persons. Individuals were selected following a multistage area probability sampling design which allowed national, sub-regional, rural and urban representativeness. A total of 23,694 participants accounted for the final study sample (2).

The survey followed the model of the determinants of active aging. It assessed seven areas: 1) economic characteristics, 2) social environment, 3) physical environment, 4) personal determinants, 5) behaviors, 6) health and social services and 7) physical measures. Physical measures were only evaluated in a subset of the sample.

Our study constituted a secondary analysis of the quantitative component of the SABE study, and utilized data from the social and economic characteristics and the health and social services sections of the dataset. The survey had four additional components: a quantitative measurement of biomarkers for cardiovascular diseases (blood glucose, hemoglobin, and lipids profile); blood pressure measurements and functionality tests in a subset of the sample; a qualitative survey for a subset of the participants which assessed their perceptions on aging and quality of life; and a quantitative component focused on caregivers which evaluated their health status and general wellbeing (1, 3-4). *Table A1* shows the distribution of the samples of the four components.

**Ethical considerations:**

The Institutional Human Ethics Committee of the University of Valle and the Bioethics Committee of the University of Caldas reviewed and approved the study (records number 09-014, O11-015, and code CBCS-021-14, respectively). The database was anonymized to protect the data privacy, so identification of individual participants was impossible.

| **Table A1:** components and participants of the SABE study | |
| --- | --- |
| **Survey component** | **Participants** |
| Quantitative study (older adults) | 23,694 |
| Biomarkers and physical measures | 4,545 |
| Qualitative component | 197 |
| Quantitative study (caregivers) | 1,141 |
|  | |

**Access to data**

The Colombian Ministry of Health (MOH) produces the SABE dataset, which is put into the public domain. Both the full data and the main reports produced from the SABE study are available to the public upon request to the email: [repositorio@minsalud.gov.co](mailto:repositorio@minsalud.gov.co). Further information can be found at: <https://www.minsalud.gov.co/sites/rid/Paginas/buscar.aspx>.

**References:**

1. Ministerio de salud y Protección Social, Departamento de Ciencia, Tecnología e Innovación - COLCIENCIAS, Universidad del Valle, Universidad de Caldas. Salud Bienestar y Envejecimiento en Colombia. Situación de persona adulta mayor. Bootá D.C., Colombia2016. p. 476.

2. Gomez F, Corchuelo J, Curcio CL, Calzada MT, Mendez F. SABE Colombia: Survey on Health, Well-Being, and Aging in Colombia-Study Design and Protocol. Current gerontology and geriatrics research. 2016;2016:7910205. PubMed PMID: 27956896. Pubmed Central PMCID: PMC5124445. Epub 2016/12/14. eng.

3. Ministerio de salud y Protección Social, Departamento de Ciencia, Tecnología e Innovación - COLCIENCIAS, Universidad del Valle, Universidad de Caldas. Encuesta SABE Colombia: Cuidado y personas cuidadoras familiares. Bogotá D.C. Colombia2016. p. 72.

4. Ministerio de salud y Protección Social, Departamento de Ciencia, Tecnología e Innovación - COLCIENCIAS, Universidad del Valle, Universidad de Caldas. Encuesta SABE Colombia: Vejez y calidad de vida en Colombia. Bogotá D.C. Colombia2016. p. 116.
