# Supplementary material for: Inequality in healthcare use among older people in Colombia
Source: Int J Equity Health. 2020 Oct 26;19:168. doi: 10.1186/s12939-020-01241-0 (PMC7646194; doi:10.1186/s12939-020-01241-0)
Supplement: Supplementary file 2 — Additional file 2. Construction of wealth index (asset index) in the study. [file 12939_2020_1241_MOESM2_ESM.docx]

**Additional file 2- Construction of wealth index (asset index) in the study**

For one of our variables to predict utilization of healthcare among older adults in Colombia, we used an asset index (AI) as a proxy for socioeconomic position. This variable allowed the participants to be classified into one of five different wealth quintiles, which were then utilized in the subsequent statistical analyses. To arrive at the AI, we followed the methodology that the Demographic Health Survey (DHS) program implements in Colombia, and thus we used the principal component analysis (PCA) with the survey weights provided in the dataset ([1-3](#_ENREF_1)).

A total of 34 variables in three categories of assets guided the construction of the index: household characteristics, ownership of physical assets and access to utilities. For household characteristics, we dichotomized eight variables: 1) Type of house (1 if participant lives in a property, 0 otherwise); 2) Type of ownership of the property (1 if the household owns the property where they live, 0 otherwise); 3) Having a separate room in the house for the participant and partner; and 4) Having a separate room to cook. The variables 5) Predominant material of house's ceiling; 6) Predominant material of house's external walls; 7) Predominant material of house's internal walls; and 8) Predominant material of house's floor, were classified as 1 if the area evaluated was from proper material, and 0 otherwise.

The physical assets evaluated were: 9) radio; 10) television; 11) stereo; 12) DVD player; 13) fan; 14) personal computer; 15) mobile phone; 16) fridge; 17) blender; 18) washing machine; 19) electrical or gas oven; 20) microwave; 21) vacuum cleaner; 22) heater; 23) air conditioning system; 24) internet connection; and 25) cable television. We dichotomized each of these variables as 1 if the household owned the asset, and 0 otherwise.

Finally, the evaluation of the household’s access to nine utilities completed the index. For the utilities, 26) electricity; 27) gas; 28) aqueduct; and 29) sewage, we created a dummy variable for each (where 1 means having access to the utility and 0 otherwise). The other utilities evaluated were: 30) main source of water used to cook or drink (1 if the house uses a public or community aqueduct as the main water source, 0 otherwise); 31) Type of toilet (1 if the toilet is connected to a sewage or waste disposal system, 0 otherwise); 32) main type of energy or fuel used to cook (1 if the house uses gas for cooking, 0 otherwise); 33) main type of lighting system (1 if the household uses an electrical lighting system, 0 otherwise); 34) main mechanism for waste disposal (1 if there's a formal waste disposal service available, 0 otherwise).

The asset index methodology provides a reliable variable for measuring socioeconomic position in settings where no income or consumption data are available ([4](#_ENREF_4)) or where they can lead to recall bias or seasonal variability. Additionally, this index is a stronger measure than the household classification by socioeconomic strata (SES) that the Colombian government uses. SES categorizes households into one of six groups (where group one is the worst-off, and group six is the best-off) based on geographic setting of the household (urban versus rural), household access to utilities and quality of infrastructure in the household’s neighborhood ([5](#_ENREF_5), [6](#_ENREF_6)). The SES measure addresses a challenge that many Latin American countries face: high rates of informal work which make standard income measures difficult to evaluate. However, the SES indicator may misclassify households as it leaves many ownership/consumption patterns without evaluation, relies only on geographic position of the household and leaves aside the living/income standards of the residents.

Many academics in the development economics sector have used and praised the AI. *Gwatkin et al* used the AI to analyze the distribution of multiple health outcomes by wealth quintile in more than 30 countries ([7](#_ENREF_7)). Likewise, *Filmer and Prichett* applied the AI to assess the relationship between wealth and educational enrollment in India, and to compare their dataset with others from India, Pakistan, Nepal and Indonesia ([8](#_ENREF_8)). In Latin America*, McKenzie* applied the AI to Mexican data to assess the relationship between school attendance and state-level inequality; and *Van der Poen* used the AI to analyze the influence of socioeconomic inequity on child malnutrition in developing countries, including nine nations from the region ([9](#_ENREF_9), [10](#_ENREF_10)).

Nevertheless, some academics criticize the AI. In a systematic review, *Howe et al* argue that an AI is a poor proxy for consumption expenditure, which is the traditional approach economists use to assess socioeconomic position ([11](#_ENREF_11)). They point out that in the studies identified, investigators only used the available asset information in datasets rather than ones based on theoretical backgrounds. Another issue critics point to is that there is no common agreement in the number of assets needed to construct the AI; therefore its creation could be somewhat arbitrary. Finally, this index may not be comparable across settings where wealth encompasses context-specific assets.

Despite its limitations, we’re confident that the AI was the best measure of socioeconomic position bearing in mind the data available. Its construction also reflected the expected heterogeneity of population wealth patterns in the study.

**References**

1. Rutstein SO. The DHS Wealth Index: Approaches for rural and urban areas. 2008.

2. Rutstein SO, Johnson K. The DHS wealth index. DHS comparative reports no. 6. Calverton: ORC Macro. 2004.

3. USAID. The DHS wealth index 2018 [19 November 2018]. Available from: https://[www.dhsprogram.com/topics/wealth-index/Wealth-Index-Construction.cfm](http://www.dhsprogram.com/topics/wealth-index/Wealth-Index-Construction.cfm).

4. Vyas S, Kumaranayake L. Constructing socio-economic status indices: how to use principal components analysis. Health policy and planning. 2006;21(6):459-68.

5. Alzate MC. La estratificación socioeconómica para el cobro de los servicios públicos domiciliarios en Colombia:¿ Solidaridad o focalización?: CEPAL; 2006.

6. Departamento Nacional de Planeación. La estratificación socioeconómica: avances y retos. República de Colombia; 1997.

7. Gwatkin DR, Rutstein S, Johnson K, Suliman E, Wagstaff A, Amouzou A. Socio-economic differences in health, nutrition, and population within developing countries. Washington, DC: World Bank. 2007:287.

8. Filmer D, Pritchett LH. Estimating Wealth Effects without Expenditure Data-or Tears: An Application to Educational Enrollments in States of India. Demography. 2001;38(1):115-32.

9. McKenzie DJ. Measuring inequality with asset indicators. Journal of Population Economics. 2005;18(2):229-60.

10. Van de Poel E, Hosseinpoor AR, Speybroeck N, Van Ourti T, Vega J. Socioeconomic inequality in malnutrition in developing countries. Bulletin of the World Health Organization. 2008;86(4):282-91. PubMed PMID: 18438517. Epub 02/19.

11. Howe LD, Hargreaves JR, Gabrysch S, Huttly SRA. Is the wealth index a proxy for consumption expenditure? A systematic review. Journal of Epidemiology and Community Health. 2009;63(11):871-7.
