# Supplementary material for: Inequality in healthcare use among older people in Colombia
Source: Int J Equity Health. 2020 Oct 26;19:168. doi: 10.1186/s12939-020-01241-0 (PMC7646194; doi:10.1186/s12939-020-01241-0)
Supplement: Supplementary file 3 — Additional file 3. Additional decomposition analyses of concentration indices. [file 12939_2020_1241_MOESM3_ESM.docx]

**Additional file 3- Additional decomposition analyses of concentration indices**

| **Table A3:** Additional decomposition analyses of concentration indices | | | | | | | | | | | | |
| --- | --- | --- | --- | --- | --- | --- | --- | --- | --- | --- | --- | --- |
|  | **Any doctor visit in the last 4 months** | | | | **Any hospitalization in the last year** | | | | **Use of pap smear in the last 2 years** | | | |
| Variable | Elasticities | CI | Contribution to CI (%) | p values | Elasticities | CI | Contribution to CI (%) | p values | Elasticities | CI | Contribution to CI (%) | p values |
| **PREDSPOSING FACTORS** |  |  |  |  |  |  |  |  |  |  |  |  |
| Age (above 65 years)¬ | 0.030 | -0.015 | -1.193% | 0.014* | 0.153 | -0.015 | 23.178% | 0.091 | -0.506 | -0.015 | 7.181% | <0.001** |
| Being male | -0.025 | -0.024 | 1.525% | 0.028* | 0.064 | -0.024 | 14.862% | 0.118 | NA | | | |
| Married/cohabiting | 0.005 | 0.055 | 0.711% | 0.737 | 0.031 | 0.055 | -16.479% | 0.586 | 0.063 | 0.055 | 3.184% | 0.001** |
| Education (Primary education or above)¬ | 0.024 | 0.138 | 8.504% | 0.454 | 0.061 | 0.138 | -82.516% | 0.589 | -0.006 | 0.138 | -0.828% | 0.953 |
| Belonging to an ethnic minority | -0.002 | -0.138 | 0.647% | 0.878 | 0.011 | -0.138 | 14.403% | 0.477 | -0.012 | -0.138 | 1.547% | 0.242 |
| Being displaced | 0.003 | -0.207 | -1.490% | 0.441 | -0.003 | -0.207 | -7.103% | 0.919 | 0.005 | -0.207 | -0.935% | 0.512 |
| **ENABLING FACTORS** |  |  |  |  |  |  |  |  |  |  |  |  |
| Wealth quintile (2nd poorest and above)¬ | 0.083 | 0.267 | 57.725% | 0.054 | -0.037 | 0.267 | 98.106% | 0.338 | 0.331 | 0.267 | 81.425% | <0.001** |
| Geographic region (living outside Bogota)¬ | 0.023 | -0.045 | -2.697% | 0.322 | 0.064 | -0.045 | 28.236% | 0.212 | 0.018 | -0.045 | -0.763% | 0.265 |
| Area of residence (urban) | 0.074 | 0.020 | 3.808% | 0.002** | 0.182 | 0.020 | -35.374% | 0.006** | 0.120 | 0.020 | 2.188% | 0.004** |
| Type of health insurance (insured)¬ | 0.368 | 0.053 | 51.001% | <0.001** | -0.069 | 0.053 | 36.223% | 0.594 | 0.381 | 0.053 | 18.739% | <0.001** |
| Receives a pension | 0.007 | 0.307 | 5.585% | 0.737 | -0.086 | 0.307 | 260.187% | 0.003** | 0.013 | 0.307 | 3.654% | 0.239 |
| **NEED FACTORS** |  |  |  |  |  |  |  |  |  |  |  |  |
| Multimorbidity (one chronic disease or more) ¬ | 0.215 | 0.044 | 24.837% | <0.001** | 0.435 | 0.044 | -189.236% | <0.001** | 0.086 | 0.044 | 3.540% | 0.058 |
| Self-rated health (good health) | -0.065 | 0.048 | -8.145% | <0.001** | -0.210 | 0.048 | 99.913% | <0.001** | 0.029 | 0.048 | 1.305% | 0.161 |
| Barthel Index (not being independent) ¬ | 0.052 | -0.011 | -1.515% | 0.237 | -0.004 | -0.011 | -0.412% | 0.011* | -0.299 | -0.011 | 3.101% | 0.237 |
| Walking impairment (having any impairment) ¬ | -0.021 | -0.021 | -2.022% | 0.031* | 0.050 | -0.021 | 10.213% | 0.681 | -0.062 | -0.021 | 1.193% | 0.188 |
| Walking help | 0.022 | 0.008 | 0.468% | 0.422 | -0.350 | 0.008 | 27.694% | 0.066 | 0.067 | 0.008 | 0.499% | 0.230 |
| Amputations | -0.003 | 0.144 | -1.080% | 0.180 | 0.013 | 0.144 | -18.820% | 0.061 | -0.002 | 0.144 | -0.224% | 0.824 |
| Number of observations | 17535 | | | | 17547 | | | | 9785 | | | |
| Total contribution (statistically significant factors) | 69.81% | | | | 135.08% | | | | 113% | | | |
| * Statistical significance at 5% level ** Statistical significance at 1% level CI: Concentration index NA: Not applicable ¬ categorical variable that was recoded as a dichotomous variable for the decomposition analysis. The text in parenthesis indicates the new category used | | | | | | | | | | | | |
